# Ameliorative effect of licorice extract against the detrimental effect of glyphosate-based pesticide: Toxicity and health

**DOI:** 10.1016/j.heliyon.2024.e31623

**Published:** 2024-05-21

**Authors:** Ahmed N. Elkattan, Sayad El-saadany, Mohamed Azzazy, Tarek M. Okda, Maha Mamdouh, Osama Ahmed, Ali H. El-Far, Manar ElKhayat, Ghadeer M. Albadrani, Muath Q. Al-Ghadi, Mohamed M. Abdel-Daim, Hala El Daous

**Affiliations:** a Institute of Graduate Studies and Environmental Research, DamanhourUniversity, 22511, Damanhour, Egypt; b Biochemistry Department, Faculty of Agriculture, Zagazig University, 44511, Zagazig, Egypt; c Plant Ecology, Institute of Desert Studies, Sadat City University, 32897, El Sadat City, Egypt; d Biochemistry Department, Faculty of Pharmacy, Damanhur University, 22511, Damanhour, Egypt; e Department of Physiology, Faculty of Veterinary Medicine, Benha University, 13736, Mushtuhur, Toukh, Qalioubia, Egypt; f Department of Anatomy and Embryology, Faculty of Veterinary Medicine, Benha University, 13736, Mushtuhur, Toukh, Qalioubia, Egypt; g Department of Biochemistry, Faculty of Veterinary Medicine, Damanhour University, 22511, Damanhour, Egypt; h Department of Bacteriology, Immunology and Mycology, Faculty of Veterinary Medicine, Benha University, 13736, Mushtuhur, Toukh, Qalioubia, Egypt; i Department of Biology, College of Science, Princess Nourah bint Abdulrahman University, 84428, Riyadh, 11671, Saudi Arabia; j Department of Zoology, College of Science, King Saud University, P.O. Box 2455, Riyadh, 11451, Saudi Arabia; k Department of Pharmaceutical Sciences, Pharmacy Program, Batterjee Medical College, P.O. Box 6231, Jeddah, 21442, Saudi Arabia; l Pharmacology Department, Faculty of Veterinary Medicine, Suez Canal University, Ismailia, 41522, Egypt; m Faculty of Veterinary Medicine, Benha University, 13736, Mushtuhur, Toukh, Qalioubia, Egypt

**Keywords:** Glyphosate-based pesticide, Toxicity, Licorice (*Glycyrrhiza glabra*), Antioxidant, Apoptosis

## Abstract

This study sheds the light on the potential of licorice (Glycyrrhiza glabra) root aqueous extract as a cornerstone for mitigating and detoxifying the residues of the widely used agricultural Glyphosate-based pesticides (GBPs). This study examined the GBPs toxic effects on kidney, liver, thyroid functions, and apoptosis using 50 adult male albino rats. All rats were divided into 5 groups, with 10 each. Control: served as untreated rats. GBP: rats were treated with 1 mL glyphosate solution 24 % orally for three weeks. The glyphosate-treated rats were gavaged with licorice root aqueous extractsolution (100, 200, and 300 mg/mLdistilled water, respectively) daily for three weeks. Licorice root aqueous extract solution (300 mg/mL distilled water) yielded notable reductions in liver, kidney enzymes, albumin, and AFP levels within the serum. Immunological tests, including immunohistochemical evaluations of caspase-3 and TNF-*α* expressions revealed a dose-dependent attenuation of apoptosis and inflammation with licorice intervention. This will provide a valuable perspective for agricultural practices future and paving the way for a more sustainable approach for using GBPs in animal agriculture industries.

## 1 Introduction

Glyphosate-based pesticides (GBPs), one of the most significant endocrine disruptors (EDs), have played a prominent role in agriculture for over four decades, primarily to control vegetation. Nevertheless, the preceding two decades has witnessed a dramatic increase in the GBPs utilization in agriculture primarily in response to the emergence of glyphosate-tolerant genetically modified crops [1]. Consequently, a growing concern has been expressed regarding the potential adverse effects of these substances on the well-being and productivity of humans and animals. In industrial food production, the testing for glyphosate residues in cereals and food products has emerged as a topic of paramount interest. The levels of GBPs levels are dose-dependent and typically increase with higher concentrations detected following increased application frequency and/or application closer to harvesting times [2]. Notably, residues of glyphosate have been identified in the liver, spleen, lungs, intestines, heart, muscles, and kidneys of commercial broilers and dairy heifers that were fed diets containing these genetically modified organisms (GMOs) [3]. Moreover, traces of GBPs residues have been detected in the blood and urine of humans, as well as the urine of dairy cows, rodents, and rabbits [4,5]. In addition, the widespread use of glyphosate resulted in the accumulation of residues in environmental settings, such as soil and water [6].

Animal and epidemiological studies have yielded substantial contributions to this field of research, shedding light on the potential adverse impacts of GBPs exposure [7]. In 2015, the classification of GBPs as "possibly carcinogenic to humans" by the WHO International Agency for Research on Cancer highlighted the urgency of understating the GBPs impacts on human and animals’ health [8]. Specifically, there is evidence that glyphosate is an endocrine disruptor, capable of affecting the production and distribution of hormones such as testosterone and estrogen, as well as the tissues that respond to them [[9], [10], [11]].Studies exploring the GBPs-induced alterations within the gonadal axis have shown that glyphosate disrupts aromatase function, leading to consequential changes in rodent reproductive development. This further demonstrates the propensity for GBPs to disrupt the endocrine system and raises questions about its broader implications [12]. In context of animal agriculture, the persistent challenge of diminishing fertility in genetic stock looms large, resulting in substantial economic losses [13]. The underlying causes of these problems are likely to be multifaceted, and so will be their solutions. Given what is known about the effects of GBPs exposure on reproductive health and the risk that GBPs exposure poses to agriculturally significant animals, it is likely that GBPsexposure is one of the causes of the progressive decline in genetic stock fertility. The question of the toxicological assessment of technical pesticides or their commercial formulations raises concerns about the endocrine-disrupting potential of glyphosate [14]. Therefore, identifying and mitigating this anticipated risk could prove helpful in ameliorating the threat that fertility losses pose to the animal agricultural industries.

While some interventions have been explored to mitigate glyphosate-based toxicity across various settings as ultra-high-pressure (UHP) and ferrous sulfate for soil [15] and physical, biological, and advanced oxidation processes for wastewater, melatonin administration for oocyte deterioration [16], and black seed dietary supplementation to improve physiological parameters in animals exposed to glyphosate [17], limitations such as potential environmental impacts and variability in effectiveness across species and different body systems exist, in addition to the need for further studies fill the gaps in terms of the interplay between these interventions and different systems, such as endocrine disruptors, liver, and kidneys.

The roots of the leguminosae plant *Glycyrrhiza glabra*, has been known as a rich source of a secondary metabolite known as licorice. Licorice is a glycoside of glycyrrhetinic acid with both therapeutic and nutritional applications. As a Phyto-additive, this plant contains numerous compounds with potential anti-cancer, anti-atherogenic, anti-diabetic, anti-asthmatic, anti-inflammatory, anti-microbial, and antispasmodic properties, including glycyrrhizin, glycyrrhizinic acid, isoliquiritin, and glycyrrhizic acid (GA) [18,19]. These components have distinct but complementary functions as hepatoprotections and immunity boosters. In addition to their antidepressant, sedative, and anticoagulant properties, they can also be used to promote hair growth and reduce adiposity. *G*. *glabra* root extract contains numerous advantageous compounds, including phytosterols, choline, and tannins. The prevention of diseases and other pathological conditions caused by *G*. *glabra* has recently been the subject of intensive research [13].

There is a limited number of published studies describing licorice-based interventions in mitigating the adverse impacts of endocrine disruptors, specifically GBPs. Consequently, this study is a sub-study of a project whose primary objective was to evaluate the effect of commercial GBPs on the liver, kidney, and thyroid functions to investigate the mechanistic role of licorice extract in modulating and potentially mitigating the adverse and toxic effects of GBPs in male rats. This will fill critical gaps in our understanding of the interplay between plant-based interventions, specifically licorice and endocrine disruptors, offering a valuable perspective for the future of agricultural practices, and paving the way to a more sustainable and informed approach to utilizing GBPs in the animal agricultural industry, assuring the continued viability and safety of this vital industry, as well as the health of animals and humans.

## 2 Materials and methods

### 2.1 Experimental animals

All animal procedures described in this study were ethically approved from both the Institutional Animal Care and Use Committee (IACUC) at Benha University, Egypt (IACUC/2/F/264/2022), and the Ethical Approval Committee of the Faculty of Veterinary Medicine, Benha University, Egypt (BUFVTM 08-04-23). In this study, fifty adult male Sprague-Dawley albino rats, weighing between 150 and 200 g, were obtained from the Animal Facility of the Faculty of Veterinary Medicine at Benha University, Egypt. Throughout the study, the rats were housed under controlled conditions, maintaining a temperature of 25 °C, 60 % humidity, and a 12-h light-dark cycle. They were provided with a standard basal diet (Table 1) and water ad libitum. In accordance to ethical standards and animal welfare guidelines, rats were acclimatized to laboratory conditions preceding experimentation to minimize stress. Housing facilities maintained optimal environmental parameters, and any procedures involving animals were performed with care to minimize pain, distress, and suffering. In addition, Continuous monitoring of animal health and well-being was conducted throughout the study, with prompt intervention and veterinary care provided when needed. Rats were euthanized by asphyxiation using CO2, followed by cervical dislocation.

**Table 1:** Ingredients of the basal diet.

Ingredients	g/kg diet
Corn flour	529.5
Casein	200
Sucrose	100
Soybean oil	70
Cellulose	50
Mineral mix	35
Vitamin mix	10
l-cystine	3
Choline	2.5

### 2.2 Drugs and chemicals

The glyphosate pesticide SL formulation (soluble liquid concentrate) used in this study was supplied by Atta Agricultural Development Company (Kufr El-Sheik, Egypt) at a concentration of 48 %. A 24 % glyphosate solution was prepared by diluting the pesticide with distilled water in a 1:1 ratio.

Licorice is widely cultivated in Middle East, Asia, and Europe [20]. In this study*G. glabra* was obtained as raw dried licorice plant with rootsfromEgyptian herbal marketplaces, Benha, Qalioubia, Egypt, and its identity and authenticity were confirmed by a botanist and pharmacologist. An aqueous extract of *G. glabra* was prepared following the previously reported procedures [21]. In brief, the dried licorice roots were ground into a fine powder and 50 g of the powder was mixed with 500 ml of distilled water and stirred magnetically at 40 °C for 24 h. The resulting suspension was filtered through Whatman No. 1 filter paper and concentrated at 40 °C under reduced pressure. Freezedrying of the water extract yielded powdered aqueousof 12.5 % extract.

### 2.3. Phytochemical analysis of licorice root extract

Phytochemical analysis of the licorice root extract included the determination of total saponin concentration using a colorimetric technique as described by Makkar et al. (2007) [22] and the results were expressed as milligrams of diosgenin equivalent per gram of material (mg DE/g). The total flavonoid content was quantified by determining the quercetin equivalent via a calibration curve following the method described by Ordoñezet al., (2006) [23]. Total phenol (TP) and total tannin (TT) concentrations were determined using the Folin-Ciocalteu method, as described by Cheel et al. (2007) [24], and expressed as milligrams of gallic acid equivalent (GAE) per gram of dried extract weight. The total terpenoid content was determined according to the method by Koleva et al. (2002) [25] and quantified as milligrams of linalool (LE) per gram of dried material.

### 2.4 Experimental design

In this study, a total of fifty rats were divided into 5 groups, each comprising 10 rats (*n* = 50). The control group received daily water and remained untreated throughout the experimental period. The glyphosate-treated (GBP) group was orally administered 1 mL of a 24 % glyphosate solution via oral gavage daily for three weeks. In addition to glyphosate treatment, the animals in three experimental groups, namely (GBP + GGE100), (GBP + GGE200), and (GBP + GGE300), were further subjected to daily oral gavage with licorice solution at concentrations of 100, 200, and 300 mg/mL distilled water (DW), respectively, over the same three-week period. Biochemical tests were conducted on the rats' blood after the first, second, and third weeks to assess the protective effects of licorice solution against the adverse effects induced by glyphosate exposure.

### 2.5 Blood sampling

Blood sampleswere collected from the tail veins of rats and subjected to centrifugation at 1500*g* for 10 min to obtain serum, which was stored at −20 °C for subsequent biochemical analysis.

### 2.6 Tissue specimens

At the end of the experiment, rats were anesthetized using isoflurane inhalation and euthanized by cervical dislocation. Liver tissue specimens were rapidly harvested, rinsed with physiological saline, and fixed in 10 % formalin for subsequent histopathological and immunohistochemical examinations.

### 2.7 Biochemical assays

#### 2.7.1 Determination of kidney function tests

Creatinine and urea levels were measured using kinetic kits (Diamond Diagnostic, Egypt) and (Beacon Diagnostics Liquizyme Urea Berthelot Method Test Kit, India), respectively, following the manufacturer's instructions.

#### 2.7.2 Determination of liver enzymes

The serum activities of alanine aminotransferase (ALT) and aspartate aminotransferase (AST) were determined using kinetic assays (MG, Egypt) according to the manufacturer's instructions.

#### 2.7.3 Determination of albumin

Serum albumin levels were measured using enzymatic colorimetric reagents (Arena Bio.Scien., Egypt) according to the manufacturer's instructions.

#### 2.7.4 Determination of serum alpha-fetoprotein (AFP)

Serum alpha-fetoprotein (AFP) levels were determined using Foresight® AFP EIA Kits (San Diego, California, USA) following the manufacturer's instructions.

#### 2.7.5 Determination of serum thyroid hormones

Serum thyroid stimulating hormone (TSH), thyroxine (T4), triiodothyronine (T3), free T4 (FT4), and free T3 (FT3) levels were determined using respective rapid quantitative tests from Xiamen Biotime Biotechnology Co., Ltd. (China) following the manufacturer's instructions.

### 2.8 Histopathological examinations

The rats’ liver samples were fixed in 10 % formalin for 48–72 h, briefly rinsed in flowing water, then dehydrated in 100 % ethyl alcohol, cleansed with xylene, embedded in paraffin, and baked at 50 °C for 24 h in a hot air oven. Paraffin-embedded tissue blocks were sectioned a sledge microtome at a thickness of 4 mm, and the sections were mounted on glass slides. Deparaffinization, Hematoxylin and eosin staining of tissue sections [26]. were performed for histological examination using a computerized light microscope (Leica DM 3000 LED).

### 2.9 Immunohistochemical evaluation of caspase-3 and TNF-α

Liver tissue samples from experimental groups were fixed in formalin, embedded in paraffin, and sectioned into 4–5 μm thick slices. Antigen retrieval, a crucial process to expose the target antigens for antibody binding, was achieved by microwave heating of tissue sections in a citrate buffer solution (pH 6.0). The sections were then exposed to thorough blocking procedures to minimize non-specific binding and quenching of endogenous peroxidase activity. Sections were then incubated with primary antibodies specific to cleaved caspase-3 and TNF-*α*. Following primary antibody incubation, sections were subjected to treatment with a secondary antibody conjugated with horseradish peroxidase (HRP). HRP activity was visualized using diaminobenzidine (DAB) chromogen, resulting in a brown precipitate at sites expressing cleaved caspase-3 and TNF-*α*. Hematoxylin counterstaining was used to provide appropriate information about cellular morphology.

The subsequent immunohistochemical evaluation of these stained sections encompassed qualitative and quantitative dimensions. Qualitative assessment of cleaved caspase-3 and TNF-*α* immunoreactivity involved visual examination under a light microscope, facilitating comparisons of staining intensities among experimental groups. Quantitative analysis utilized Image J software (version 1.36, NIH, Bethesda, MD, USA)to meticulously quantify expression percentages. This involved examining five randomly selected fields from each liver section to ensure comprehensive and representative assessment of staining patterns.

### 2.10 Molecular docking assessment

The three-dimensional (3D) structures of the following proteins: albumin (P02770), thyroxine-binding globulin (P35577), transthyretin (PDP: 1IE4), caspase-3 (P55213), tumor necrosis factor receptor superfamily member 1A (TNFRSF1A; P22934), and tumor necrosis factor receptor superfamily member 1B (TNFRSF1B; Q80WY6) were retrieved from the AlphaFold Protein Structure Database (https://alphafold.ebi.ac.uk/) and the RCSB Protein Data Bank (RCSB PDB; https://www.rcsb.org/). Additionally, the 3D structures of licorice's bioactive compounds were retrieved from the LOTUS database (https://lotus.naturalproducts.net/), a database for natural products.

Proteins were prepared for docking using the Molecular Operating Environment (MOE; Montreal, QC, Canada) software. The 3D structure of TQ was retrieved from the PubChem database. Finally, MOE software was used for molecular docking, analysis of protein-ligand interactions, and visualization.

### 2.11 Statistical analyses

The Shapiro-Wilk test was used to assess the normality of data distribution. To analyze the interaction between groups and time, a Two-Way Repeated Measures ANOVA with Geisser-Greenhouse's epsilon (*p-*value<0.05) was used. Post-hoc Dunnett's multiple comparisons tests were conducted to compare different groups within specific time intervals (Adjusted *p-*value<0.05). These statistical analyses were performed using GraphPad Prism 10.0.2 software.

Furthermore, the results of quantitative immunohistochemical evaluations were subjected to statistical examination. Robust one-way ANOVA tests were conducted to determine the statistical significance of differences in expression levels observed among the treatment groups (*p-*value<0.05). Post-hoc Tukey's tests were conducted to provide additional insights. Statistical analysis was performed using the SPSS software for Windows (version 20.0; SPSS Inc., Chicago, IL, USA).

## Ethical approval

The experimental protocol was approved by the Institutional Animal Care and Use Committee (IACUC), Benha University, Egypt (IACUC/2/F/264/2022). Additionally, this study was approved by the Ethical Approval Committee, Faculty of Veterinary Medicine, Benha University, Egypt (BUFVTM 08-04-23).

## 3 Results

### 3.1 Phytochemical analysis of licorice root extract

Phytochemical analysis of *G. glabra* root extract revealed the presence of various bioactive components. Notably, the investigated extract displayed a relatively high content of total saponin, with a measured value of 201.24 ± 1.68 mg diosgenin per gram of dry weight. Furthermore, the extract exhibited significant quantities of other key phytochemicals, including total flavonoids estimated at 25.45 ± 0.23 mg quercetin per gdry weight, and total phenols at 50.52 ± 0.26 mg gallic acid equivalents per gram dry weight. Additionally, total tannins were estimated at 7.93 ± 0.11 mg gallic acid equivalents per gdry weight. The extract also contained total terpenoids at 12.62 ± 0.25 mg linalool per gdry weight(Table 2).

**Table 2:** Bioactive chemical components of licorice (*Glycyrrhiza glabra)* root extract.

Component	Values^a^
Total saponin	201.24 ± 1.68 mg diosgenin/g dry weight
Total flavonoids	25.45 ± 0.23 mg quercetin/g dry weight
Total phenols	50.52 ± 0.26 mg gallic/g dry weight
Total tannins	7.93 ± 0.11 mg gallic/g dry weight
Total terpenoids	12.62 ± 0.25 mg linalool/g dry weight

a Values are given as mean (*n* = 5) standard deviation (absolute value).

### 3.2 Biochemical assays

**Fig. 1:**
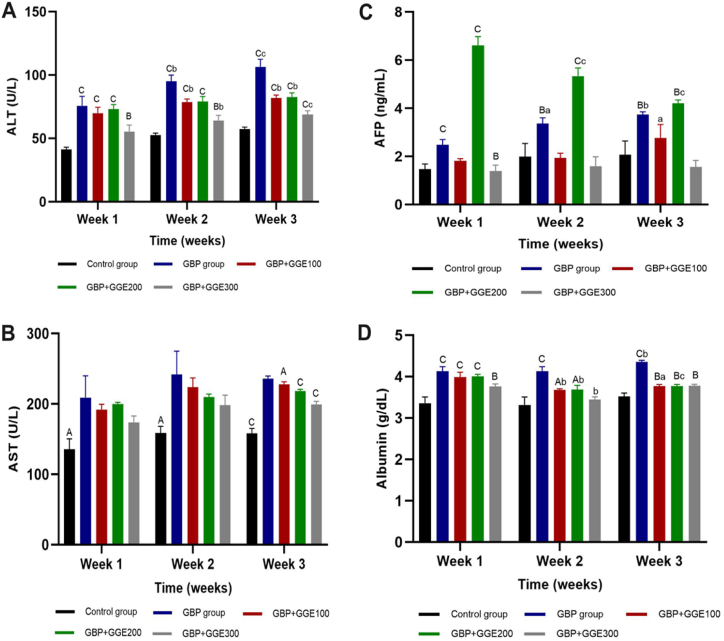
The effect of licorice administrationon GBP treated rats'liver functions, (A, ALT (U/L)), (B, AST (U/L)), (C, AFP (ng/mL))(D,albumin (g/dL)), within 1st, 2nd, and3^rd^ week of experiment. Capital letters are significance of each group vs control negative group within that time point, and small letters are significance of each treatment in week 2 and week 3 vs week 1. Control group: Rats served as controls by receiving water daily and being left untreated. GBP group: The animals were administered glyphosate solution 24 % (1 mL) orally directly into the buccal cavity of rats by gavage daily for three weeks. The glyphosate-treated animals in (GBP + GGE100), (GBP + GGE200), and (GBP + GGE300) were gavaged with licorice solution (100, 200, and 300 mg/mLDW, respectively) daily for three weeks.

The results of this study reveal significant variations in numerous measured parameters throughout different time intervals and between various experimental groups. Except for AFP and T4, all measured parameters exhibited substantial differences during the experiment. Additionally, significant differences were observed between the tested groups across all evaluated parameters. Notably, the interaction between time and groups also displayed significant differences for all measured parameters, except for AST and T4.Analysis of liver enzymes (ALT and AST) and albumin levels consistently showed substantially higher values in all tested groups compared to the control negative group across all time intervals. Meanwhile, AFP levels exhibited significant elevation only in the GBP group and (GBP + GGE100) in comparison to the control group (Fig. 1 A, B, C, and **D**).

**Fig. 2:**
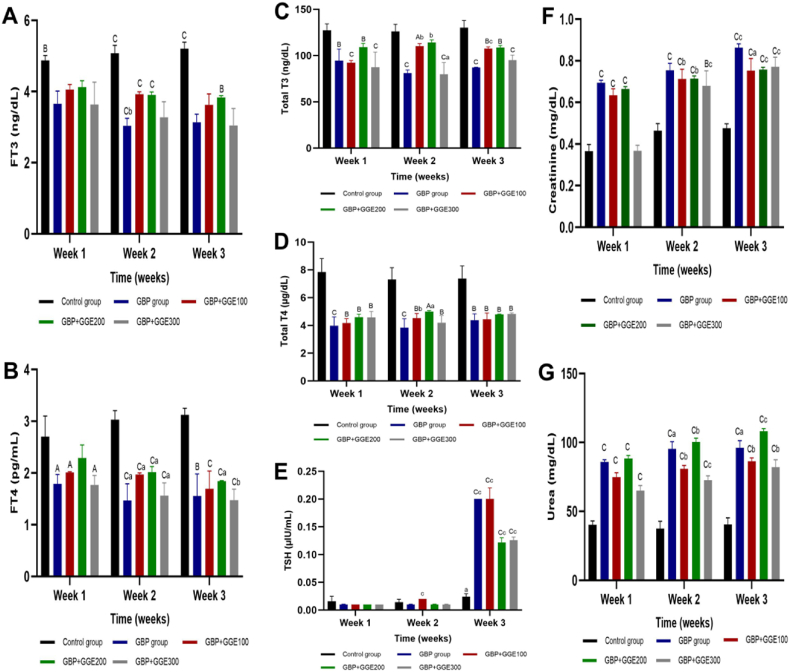
The effect of licorice administrationon GBP treated rats'thyroid hormones and kidney functions, (A, FT3 (ng/dL)), (B, T3 (ng/dL)), (C, T4 (μg/dL))(D, FT4 (Pg/dL)), (E, TSH ((μU/mL)(F, creatinine (mg/dL)) and (G, urea (mg/dL)), 1st, 2nd, 3rd week of experiment. Capital letters are significance of each group vs control negative group within that time point, and small letters are significance of each treatment in week 2 and week 3 vs week 1Control group: Rats served as controls by receiving water daily and being left untreated. GBP group: The animals were administered glyphosate solution 24 % (1 mL) orally directly into the buccal cavity of rats by gavage daily for three weeks. The glyphosate-treated animals in (GBP + GGE100), (GBP + GGE200), and (GBP + GGE300) were gavaged with licorice solution (100, 200, and 300 mg/mLDW, respectively) daily for three weeks.

Our findings in the context of thyroid function revealed a significant decrease in FT3 levels across all experimental groups when compared to the control group throughout the study, with FT4 also decreasing significantly in all groups except (GBP + GGE200) after the 1st week. Additionally, T3 and T4 levels were significantly lower in all examined groups in comparison to the control negative. TSH did not display substantial differences during the 1st and 2nd weeks but exhibited a considerable increase during the 3rd week in comparison to the control group (Fig. 2 A, B, C, D, and **E**).

According to kidney function tests, the GBP group, (GBP + GGE100), and (GBP + GGE200) consistently exhibited significantly elevated serum creatinine levels compared to the control negative group over the entire investigation. In contrast, (GBP + GGE300) displayed a significant increase in serum creatinine levels only during the 2nd and 3rd weeks. Furthermore, serum urea consistently demonstrated a significant difference between the control negative group and all other experimental groups throughout all time periods (Fig. 2 F and **G**).

In short, the outcomes of this study revealed highly significant differences between various experimental groups across different time intervals for urea, creatinine, albumin, ALT, AST, TSH, FT3, and FT4. Conversely, there were no significant differences in AFP and T4 over time. The interaction between time and group was non-significant for T4 and AST.

### 3.3 Histopathological assessment

**Fig. 3:**
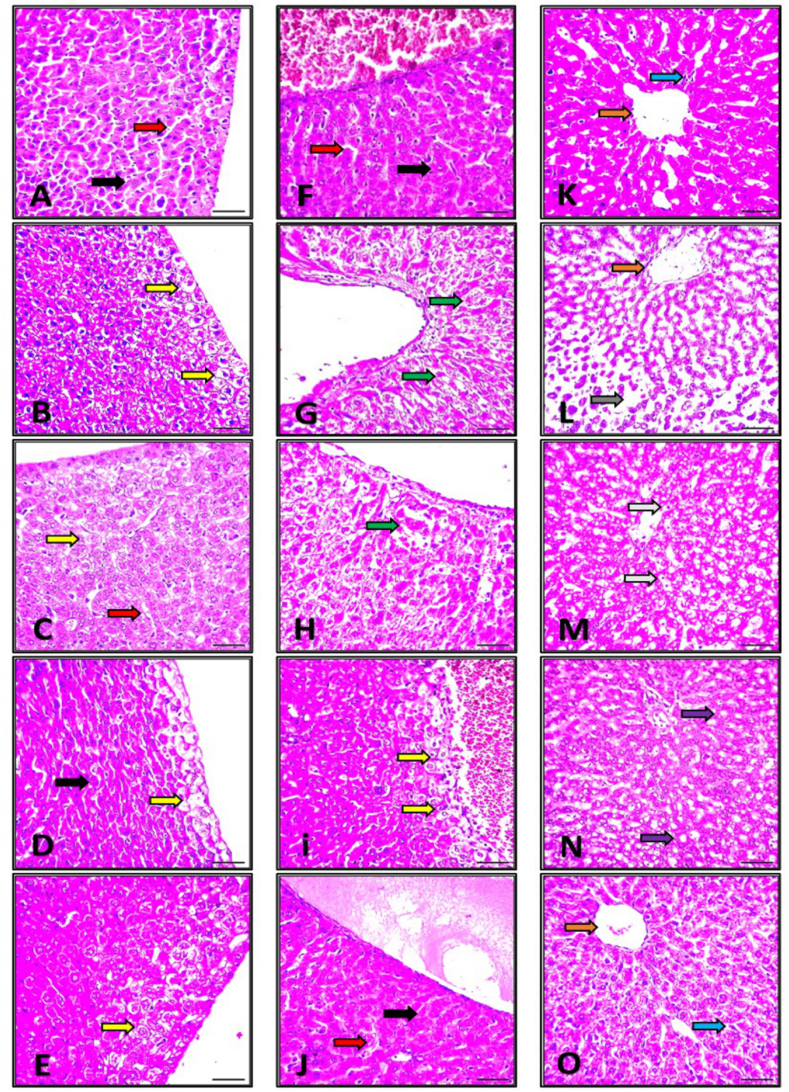
Histopathological sections of rats' livers exposed to a sub-lethal dose of glyphosate -pesticide and treated with licorice supplements. A illustrates the liver of the control group, demonstrating a normal histological appearance characterized by the presence of normal hepatic cells (black arrow) and blood sinusoids (red arrow). B, liver sections from animals treated with glyphosate display noticeable features such as swelling, vacuolation, and hepatocyte damage (yellow arrow). Panels C–E further demonstrate the liver sections of Licorice-treated animals at lower, middle, and high doses, highlighting a dose-dependent decrease in the severity of pathological alterations; however, some degenerative changes as hydropic changes could be seenafter 1st week. F Illustrates the liver of the control group, demonstrating a normal histological appearance characterized by radially arranged polyhedral hepatocytes forming cords around the central vein and sinusoids. G liver sections from animals treated with glyphosate display noticeable necrosis of centrilobular hepatocytes, dissolution (green arrow), and severe hepatocyte damage (yellow arrow). Panels H-Jfurther demonstrates the liver sections of Licorice-treated animals at lower, middle, and high doses, highlighting the approximate regain of the normal appearance of hepatocytes after 2nd week.K illustrates the liver of the control group, demonstrating a normal histological appearance characterized by the presence of Kupffer cells (blue arrow), central vein (white arrow), hepatic cells, and blood sinusoids. L liver sections from animals treated with glyphosate display noticeable features such as pyknotic nuclei (black arrow), necrosis (red arrow), dilatation in blood sinusoids, activated Kupffer cells, and fatty change. Panels M − O further demonstrates the liver sections of Licorice-treated animals at lower, middle, and high doses, highlighting a decrease in the severity of pathological alterations; however, some degenerative changes as fatty change (yellow arrow) and hydropic degeneration (green arrow), could be seen after 3rd week of experiment.

In this study, we investigated the potential protective effects of licorice supplementation against liver damage induced by sub-lethal doses of glyphosate pesticide. Histological assessments of liver sections revealed significant insights into the protective benefits of licorice. The control group exhibited a typical histological pattern characterized by cords of radially organized polyhedral hepatocytes encircling the sinusoids and central vein (Fig. 3A–F, and **K**). In contrast, the positive control group exposed to glyphosate displayed considerable, mild to severe histological abnormalities in the subcapsular region of their livers, including hydropic degeneration of the liver cells and issues with the radiating structure (Fig. 3B). However, the administration of licorice supplements at varying doses resulted in a remarkable restoration of normal hepatocyte appearance.

Additionally, examination of liver sections from glyphosate-exposed rats revealed significant changes in the pericentral zone, including focal necrosis in centrilobular hepatocytes (Fig. 3G and **L**). However, these alterations were barely noticeable when licorice supplementation was present after glyphosate-induced hepatic damage, especially when a high dose was used. Alterations in the periportal zone, which showed specific clinical characteristics such as swelling, pyknotic nuclei, necrosis, dilatation in blood sinusoids, activated Kupffer cells, fatty alterations, and hepatocyte destruction, were also alarming. The liver sections from the licorice-treated rats were then examined at various time intervals (Fig. 3C-O). Notably, licorice treatment resulted in a decrease in the degree of pathological changes. Licorice supplementation specifically decreased hepatocyte damage, hazy edema, pyknotic nuclei, necrosis, dilatation in blood sinusoids, and swelling.

### 3.4 Immunohistochemical assessments

#### 3.4.1 Assessment of caspase-3 expression

**Fig. 4:**
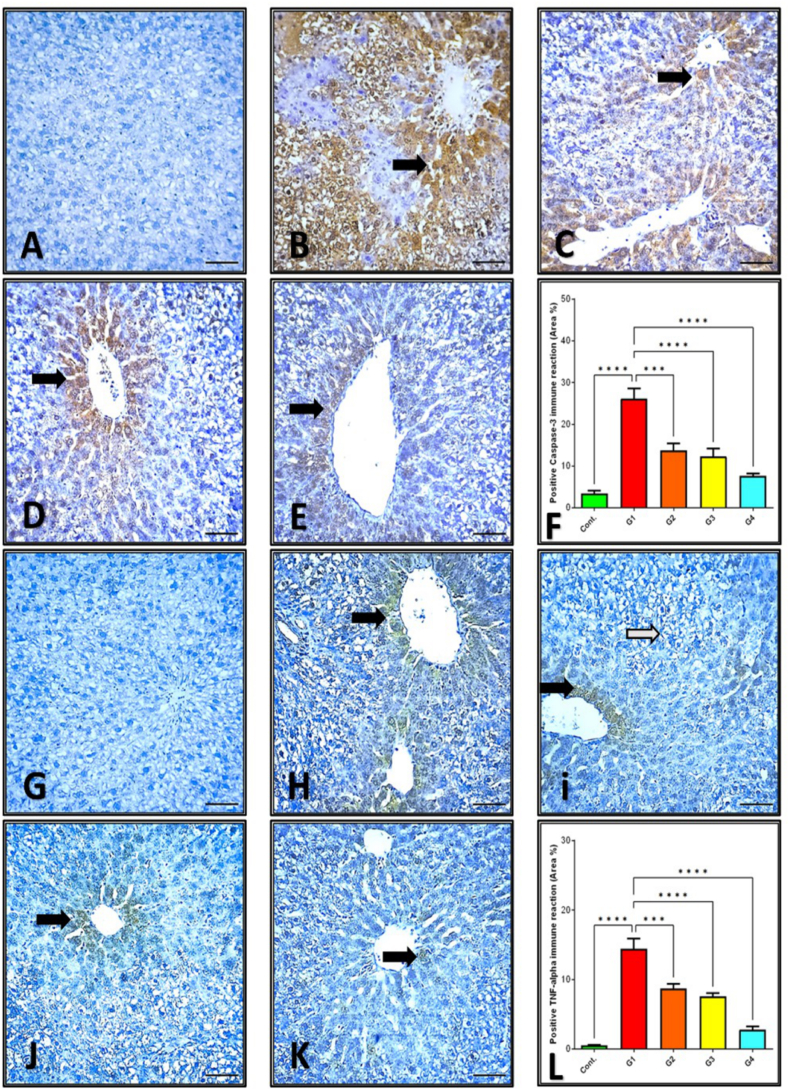
Cleaved caspase-3 Immunoreactivity in Rat Liver Sections (A–F). A Illustrates the absence of cleaved caspase-3 in the Control group. Immunohistochemical analysis of TNF-*α* Expression in Rat Liver Sections. Panel a depicts minimal TNF-*α* immunoreactivity in the Control group. Panel b shows pronounced hepatic TNF-*α* expression in the GBP group post-glyphosate treatment. B Following glyphosate treatment, pronounced cleaved caspase-3 expression was demonstrated in the GBP group. C Depicts strong immune reactivity in liver tissues from (GBP + GGE100).D and E represent liver sections from (GBP + GGE200), and (GBP + GGE300), showing a gradient from moderate to weak cleaved caspase-3 expression. F Quantifies the immunostaining area (%) of cleaved caspase-3 expression, with data presented as mean ± SE; a distinct asterisk indicates significant differences. Arrows highlight the specific areas of immune expression. Immunohistochemicalanalysis of TNF-*α* Expression in Rat Liver Sections (G–L). Panel Gdepicts minimal TNF-*α* immunoreactivity in the Control group. Panel H shows pronounced hepatic TNF-*α* expression in the GBP group post-glyphosate treatment. Panels I, J, and K represent liver tissues from (GBP + GGE100), (GBP + GGE200), and (GBP + GGE300), respectively, illustrating a gradient from moderate to weak TNF-*α* expression. For comparative purposes, Panel L presents the quantification of the immunostaining area (%) for cleaved caspase-3 expression, with data expressed as the mean ± standard error (SE) and distinct superscript asterisk denoting statistically significant differences. Brown staining denotes TNF-*α* positivity.

The evaluation of apoptosis, a tightly regulated cellular death mechanism, was conducted through immunohistochemical staining for cleaved caspase-3 in liver tissue sections. In the control group, liver samples exhibited negligible caspase-3 immunoreactivity, indicative of minimal apoptotic activity (Fig. 4A). In stark contrast, the liver tissues from the GBP group, which experienced glyphosate intoxication, displayed prominent caspase-3 staining, indicating heightened apoptotic events (Fig. 4B). Similarly, (GBP + GGE100) also showed noticeable caspase-3 staining (Fig. 4C). Interestingly, rats of (GBP + GGE200) group displayed a moderate caspase-3 staining intensity (Fig. 4D). Remarkably, (GBP + GGE300), subjected to the highest licorice solution concentration (300 mg/mLDW), exhibited the most substantial reduction in caspase-3 expression among the licorice-intervened groups. However, it's essential to note that even with this significant reduction, caspase-3 expression remained elevated compared to the control group(Fig. 4E).

Quantitative analysis of cleaved caspase-3 apoptotic protein expression, computed as the percentage of stained area, supported these qualitative findings, and further underscored the modulatory effects of licorice on glyphosate-induced apoptosis in rat liver tissues. Specifically, the percentage of cleaved caspase-3 staining was 3.39 ± 1.44 % in the control group, 26.16 ± 5.93 % in the GBP -treated group, 13.48 ± 4.11 % in GBP + GGE100vs. GBP group, 12.26 ± 4.12 % in GBP + GGE200 vs. GBP group, and 7.65 ± 1.83 % in GBP + GGE300vs. GBP group. These findings demonstrate a dose-dependent attenuation of glyphosate-induced apoptotic cell death in the rat liver due to licorice intervention (Fig. 4F).

#### 3.4.2 Assessment of TNF-α expression

Assessment of TNF-*α* expression in rat liver tissues revealed varying levels of expression across different groups. The control group exhibited minimal TNF-*α* expression (Fig. 4G). In contrast, the GBP group displayed a significant increase in TNF-*α* expression (Fig. 4H). Treatment with different dosages of a 10 % licorice solution resulted in varying TNF-*α* expression levels. Specifically, GBP + GGE100, showed attenuated TNF-*α* levels (Fig. 4I). However, these levels remained elevated compared to the baseline established by the control group. Similarly, GBP + GGE200 displayed a further reduction in TNF-*α* levels but remained higher than the control group (Fig. 4J). Notably, GBP + GGE300, subjected to the highest licorice dosage (300 mg/mLDW), exhibited the most significant reduction in TNF-*α* expression among the licorice-treated groups(Fig. 4K).

The cleaved TNF-*α* expression levels were quantitatively determined by analyzing the stained regions' percentage representations. The quantitative analysis of cleaved TNF-*α* expression reinforced the modulatory ability of licorice in counteracting glyphosate-induced liver tissue anomalies, as the results confirmed significant variations across different treatment groups. The GBP group had a substantial 15.4-fold increase in TNF-*α* expression compared to the Control group. Furthermore, licorice-administered groups (GBP + GGE100, GBP + GGE200, and GBP + GGE300) displayed a notable reduction in TNF-*α* expression relative to the GBP group, highlighting the dose-dependent therapeutic efficacy of licorice. The highest licorice dosageGBP + GGE300, demonstrated the most significant reduction, achieving a 3.5-fold decrease in TNF-*α* expression compared to the GBP group. However, it's important to note that even with the highest licorice dosage, TNF-*α* level did not return to the baseline levels observed in the untreated control group (Fig. 4L).

### 3.5 Molecular docking assessment

**Fig. 5:**
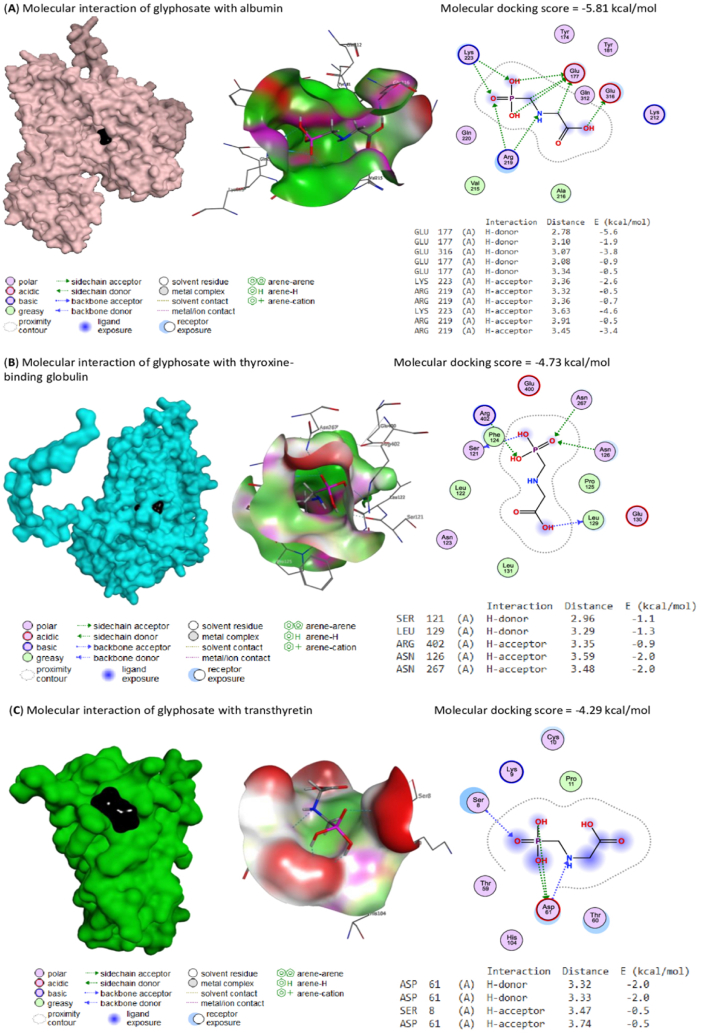
**(A)** Molecular docking interaction of glyphosate with rats' albumin. The Molecular docking score is −5.81 kcal/mol. Glyphosate interacted by H-donor bonds with GLU177 (4) and GLU316 and H-acceptor bonds with LYS223 (2) and ARG219(4) residues in the albumin's binding site. **(B)** Molecular docking interaction of glyphosate with rats' thyroxine-binding globulin. The molecular docking score is −4.73 kcal/mol. Glyphosate interacted by H-donor bonds with SER121 and LEU129 and H-acceptor bonds with ARG402, ASN126, and ASN267 residues in the thyroxine-binding globulin's binding site. **(C)** Molecular docking interaction of glyphosate with rats' transthyretin. The molecular docking score is −4.29 kcal/mol. Glyphosate interacted by H-donor bonds with ASP61(2) and H-acceptor bonds with SER8 and ASP61 residues in the transthyretin's binding site.

Molecular interactions of glyphosate with rats’ albumin, thyroxine-binding globulin, and transthyretin are represented in Fig. 5A and B, and **C**, respectively, where glyphosate bound with their binding sites with −5.81, −4.73, and −4.29 kcal/mol, respectively.

**Fig. 6:**
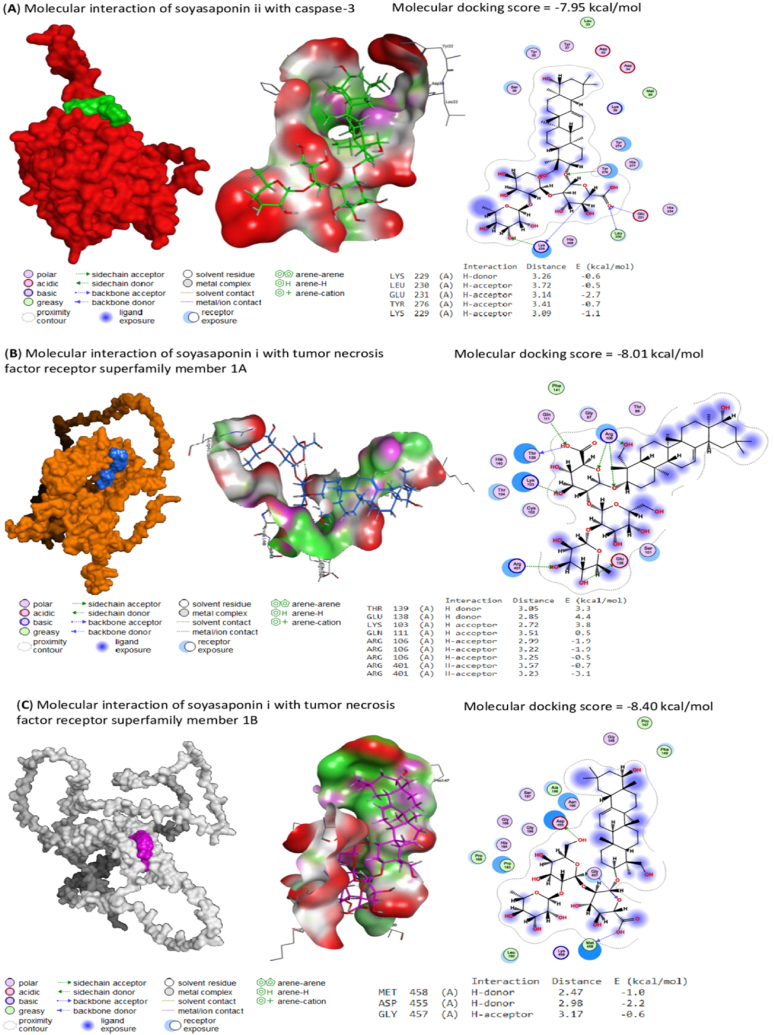
(A) Molecular docking interaction of soyasaponin ii with rats' caspase-3. The molecular docking score is −7.95 kcal/mol. Soyasaponinii interacted by H-donor bonds with LYS229 and H-acceptor bonds with LEU230, GLU231, TYR276, and LYS229 residues in caspase-3's binding site. (B) Molecular docking interaction of soyasaponin i with rats' tumor necrosis factor receptor superfamily member 1A (TNFRSF1A). The molecular docking score is −8.01 kcal/mol. Soyasaponini interacted by H-donor bonds with THR139 and GLU138 and H-acceptor bonds with LYS103, GLN111, ARG106(3), and ARG401. (C) Molecular docking interaction of soyasaponin i with rats' tumor necrosis factor receptor superfamily member 1B (TNFRSF1B). The molecular docking score is −8.40 kcal/mol. Soyasaponini interacted by H-donor bonds with MET458 and ASP455 and H-acceptor bond with GLY457 residues in TNFRSF1B's binding site.

Licorice's bioactive compounds interacted by different binding energies with the binding sites of caspase-3, TNFRSF1A, and TNFRSF1B are present in Table 3. In Fig. 6A, soyasaponin ii bound with rats' caspase-3 by binding energy of −7.95 kcal/mol. Soyasaponin ii interacted by H-donor bonds with LYS229 and H-acceptor bonds with LEU230, GLU231, TYR276, and LYS229 residues in caspase-3's binding site. In addition, soyasaponin i interacted with rats' TNFRSF1A by molecular docking score of −8.01 kcal/mol. Soyasaponin i interacted by H-donor bonds with THR139 and GLU138 and H-acceptor bonds with LYS103, GLN111, ARG106 (3), and ARG401(2) residues in TNFRSF1A's binding site (Fig. 6B). Also, soyasaponin I bound with the binding site of rats' TNFRSF1B by H-donor bonds with MET458 and ASP455 and H-acceptor bond with GLY457 residues in TNFRSF1B with binding energy of −8.40 kcal/mol (Fig. 6C).

**Table 3:** Molecular docking scores of licorice's bioactive compounds against rats' caspase-3, tumor necrosis factor receptor superfamily member 1A (TNFRSF1A), and tumor necrosis factor receptor superfamily member 1B (TNFRSF1B).

LotusID	Compounds	No.	Molecular docking scores(kcal/mol)			
			Caspase-3	TNFRSF1A	TNFRSF1B	
LTS0135577	3′-methoxyglabradin	1	−5.88	−5.50	−6.35	
LTS0158119	3-isothujone	2	−4.46	−4.50	−4.34	
LTS0032845	Rutin	3	−7.53	−6.58	−8.10	
LTS0200538	4′-*o*-methylglabridin	4	−6.02	−5.82	−6.14	
LTS0251224	5-deoxyflavanone	5	−4.76	−4.89	−5.15	
LTS0014786	6,8-diprenylgenistein	6	−6.53	−6.13	−6.52	
LTS0182567	(−)-lavandulol	7	−4.67	−4.94	−4.81	
LTS0072900	(−)-naringenin	8	−5.18	−5.28	−5.67	
LTS0191687	(−)-vestitol	9	−5.11	−5.26	−5.29	
LTS0225956	Abyssinone ii	10	−5.87	−5.57	−5.66	
LTS0275427	Afrormosin	11	−5.34	−5.44	−5.83	
LTS0044471	Amylfuran	12	−4.41	−4.37	−4.64	
LTS0068303	Asahina	13	−5.37	−4.99	−5.66	
LTS0249588	Astragalin	14	−6.28	−6.28	−6.96	
LTS0012882	Carvacrol	15	−4.45	−4.46	−4.49	
LTS0181568	Cymene	16	−4.28	−4.50	−4.26	
LTS0069837	Cynaroside	17	−6.58	−6.99	−7.08	
LTS0138668	Echinatin	18	−4.76	−5.34	−5.50	
LTS0222995	Enoxolone	19	−5.47	−5.07	−6.50	
LTS0180128	Euchrenone a5	20	−6.00	−6.18	−6.54	
LTS0048628	Euchrestaflavanone a	21	−6.04	−6.49	−6.98	
LTS0126716	Fenchone	22	−4.02	−4.53	−4.32	
LTS0073369	Formononetin 7-o-glucoside	23	−6.53	−6.63	−6.61	
LTS0082756	Formononetin	24	−5.30	−5.18	−5.62	
LTS0210648	Galangin	25	−5.07	−4.81	−5.50	
LTS0094683	Gancaonin f	26	−5.70	−5.85	−6.08	
LTS0003159	Gancaonin g	27	−5.71	−5.60	−6.09	
LTS0077774	Gancaonin h	28	−6.40	−6.10	−6.71	
LTS0072777	Gancaonin l	29	−5.65	−5.69	−6.12	
LTS0106538	Genistein	30	−5.07	−5.09	−5.15	
LTS0250433	Glabranin	31	−5.46	−5.58	−6.01	
LTS0232975	Glabrene	32	−5.32	−5.62	−5.84	
LTS0075616	Glabridin	33	−5.49	−5.57	−5.71	
LTS0151626	Glabrocoumarin	34	−5.72	−5.82	−5.66	
LTS0262018	Glabrocoumarone a	35	−5.37	−5.48	−5.98	
LTS0274460	Glabrocoumarone b	36	−5.29	−5.48	−5.77	
LTS0164961	Glabrol	37	−6.09	−6.62	−6.67	
LTS0075204	Glabrone	38	−5.49	−5.70	−6.02	
LTS0186848	Glycycoumarin	39	−6.16	−6.38	−6.49	
LTS0087818	Glycyrin	40	−5.89	−6.02	−6.62	
LTS0198644	Glycyrrhetinic acid	41	−5.90	−5.25	−6.27	
LTS0193131	Glycyrrhisoflavanone	42	−6.16	−6.17	−6.55	
LTS0121878	Glycyrrhizin	43	−7.33	−6.65	−8.00	
LTS0090907	Glyinflanin a	44	−6.73	−6.59	−7.05	
LTS0133651	Glyinflanin b	45	−5.68	−5.72	−6.03	
LTS0241667	Glyinflanin g	46	−6.48	−6.41	−6.53	
LTS0179228	Guaiacol	47	−4.02	−4.13	−4.18	
LTS0267683	Hispaglabridin a	48	−6.20	−6.16	−7.04	
LTS0155248	Hispaglabridin b	49	−5.88	−5.91	−6.52	
LTS0257369	Hydroxywighteone	50	−5.23	−5.14	−5.73	
LTS0223233	Isobavachromene	51	−5.67	−5.44	−5.90	
LTS0066952	Isoglycycoumarin	52	−6.21	−6.17	−6.29	
LTS0264727	Isolicoflavonol	53	−5.59	−5.78	−6.04	
LTS0051422	Isoliquiritin	54	−6.47	−6.56	−6.62	
LTS0254337	Isoquercetin	55	−6.39	−6.31	−6.74	
LTS0087575	Isorhamnetin 3-galactoside	56	−6.86	−6.04	−6.85	
LTS0137002	Isorhamnetin 3-o-glucoside	57	−6.11	−6.47	−7.42	
LTS0157117	Isoschaftoside	58	−6.41	−6.12	−7.21	
LTS0035187	Isovitexin	59	−5.97	−6.19	−6.73	
LTS0075703	Kanzonol b	60	−5.78	−5.91	−6.36	
LTS0266469	Kanzonol c	61	−6.13	−6.49	−6.57	
LTS0012990	Kanzonol d	62	−5.62	−5.56	−6.15	
LTS0138968	Kanzonol y	63	−6.28	−6.43	−6.91	
LTS0018267	Kumatakenin	64	−5.26	−5.55	−6.02	
LTS0106634	Licoagrochalcone a	65	−5.74	−6.13	−6.17	
LTS0020333	Licoagrochalcone b	66	−5.75	−5.72	−5.92	
LTS0187725	Licoagrochalcone c	67	−5.58	−5.93	−6.03	
LTS0270336	Licoagrochalcone d	68	−6.09	−6.40	−6.32	
LTS0018907	Licochalcone a	69	−5.58	−5.91	−5.90	
LTS0192338	Licochalcone b	70	−5.23	−5.46	−5.53	
LTS0183214	Licochalcone c	71	−5.82	−5.99	−5.99	
LTS0132019	Licocoumarone	72	−5.72	−6.03	−5.92	
LTS0244117	Licoflavanone	73	−5.76	−5.65	−6.38	
LTS0004664	Licoflavone a	74	−5.65	−5.86	−5.95	
LTS0122155	Licoflavone b	75	−6.39	−6.36	−7.00	
LTS0219719	Licoflavonol	76	−5.47	−5.92	−6.18	
LTS0263391	Licoisoflavone a	77	−5.70	−6.14	−6.09	
LTS0055944	Licoisoflavone b	78	−5.82	−5.81	−6.26	
LTS0048734	Licopyranocoumarin	79	−6.02	−6.09	−6.40	
LTS0274337	Licoricidin	80	−6.31	−6.95	−6.97	
LTS0132318	Licuroside	81	−7.19	−7.43	−7.43	
LTS0188438	Liquiritinapioside	82	−7.33	−7.20	−8.09	
LTS0103894	Liquiritin	83	−5.99	−6.33	−6.71	
LTS0142270	Liquorice	84	−7.28	−7.61	−7.95	
LTS0211446	Lupalbigenin	85	−6.14	−5.82	−6.71	
LTS0256952	Lupeol	86	−5.48	−5.52	−5.96	
LTS0229079	Lupiwighteone	87	−5.75	−5.77	−5.84	
LTS0261149	Medicarpin, (−)-	88	−5.17	−5.39	−5.57	
LTS0215385	Morachalcone a	89	−6.47	−6.09	−6.45	
LTS0202475	Myrtenal	90	−4.32	−4.58	−4.47	
LTS0031098	Naringenin	91	−4.95	−4.92	−5.54	
LTS0089772	Neoliquiritin	92	−6.17	−6.54	−6.50	
LTS0237730	Odoratin	93	−5.79	−5.50	−5.55	
LTS0235553	Ononin	94	−6.15	−6.98	−6.80	
LTS0014950	Paeonol	95	−4.51	−4.50	−4.47	
LTS0124936	Parvisoflavone b	96	−5.74	−5.98	−6.35	
LTS0151338	Phaseol	97	−5.61	−5.63	−6.19	
LTS0010732	Pinit	98	−4.42	−4.93	−4.92	
LTS0194724	Pinitol	99	−4.47	−4.79	−4.87	
LTS0141508	Pinocembrine	100	−4.97	−4.88	−5.26	
LTS0261766	Prunetin	101	−5.20	−5.42	−5.49	
LTS0119297	Pseudoionone	102	−4.80	−4.89	−4.87	
LTS0186298	Quercitrin	103	−6.45	−6.86	−6.86	
LTS0104338	Schaftoside	104	−6.89	−6.86	−7.55	
LTS0128805	Shinflavanone	105	−5.74	−6.69	−7.22	
LTS0058527	Sophoraflavanone b	106	−5.70	−6.03	−6.13	
LTS0182499	Soyasaponin i	107	−7.43	−8.01	−8.40	
LTS0184048	Soyasaponin ii	108	−7.95	−7.54	−8.23	
LTS0152081	Talmon	109	−3.90	−4.07	−4.07	
LTS0027534	Tephrinone	110	−5.98	−5.69	−6.13	
LTS0168527	Thymol	111	−4.67	−4.45	−4.46	
LTS0267055	Trifolin	112	−6.10	−6.15	−6.50	
LTS0181160	Vicenin 2	113	−7.17	−6.98	−7.54	
LTS0136408	Wighteone	114	−5.85	−5.77	−6.15	
LTS0139725	Xambioona	115	−5.92	−6.55	−6.40	
LTS0063487	Yinyanghuo d	116	−5.79	−5.57	−6.13

## 4 Discussion

The glyphosate pesticide use in agriculture has increased over the past two decades due to genetically altered glyphosate-tolerant (GT) crops [1]. Thus, concerns about these medications' long-term effects on human and animal performance and output have grown. Glyphosate residues in crops and food are important in industrial food production. Residue levels depend on dosage and often rise with frequency and closeness to harvest [2]. Glyphosate residue has been found in the liver, spleen, lungs, intestines, heart, muscles, and kidneys of commercial broilers and dairy cows fed glyphosate-tolerant (GT) crops [3]. The residue has been found in human blood, dairy cows, rodents, and rabbits' urine [5]. Large-scale glyphosate use has increased soil and water residues [6].

*Glycyrrhiza glabra*, often known as licorice, is one of the earliest and most well-known medicinal plants in use today. This study's chemical analysis of licorice root extract confirmed the presence of various active secondary metabolites. Phenols, flavonoids, tannins, saponins, and terpenoids were among those found. Our Results coincided with what previously reported as 52.1 mg gallic/g of total phenolics, and 14.8 mg RE/g of total flavonoids were found in licorice root [27]. Additionally, the active components of licorice, which include glycyrrhizin, glabridin, liquiritin, glycyrrhizic acid (GL), and glycyrrhetinic acid (GA), are among the principal biologically active substances found in the herb [28]. Licorice also contains significant amounts of triterpene saponins. Licorice has recently been found to have around seventy-seven different types of triterpenes saponins [29].

This study observed a considerable increase in liver, kidney, albumin, and AFP parameters at GBPs, the control positive group which also led to disruptions in the levels of thyroid hormones. The presence of histopathological findings substantiated the occurrence of liver cell injury, hence offering supplementary evidence about these observed effects. The findings have been corroborated by another report which has reported that prolonged exposure to glyphosate resulted in notable changes in blood parameters indicative of hepatorenal impairment, as well as minor histomorphology irregularities in the organs [30]. The occurrence of hepatotoxicity resulting from glyphosate exposure is associated with the release of intracellular enzymes, specifically serum ALT and AST, into the bloodstream because of cellular membrane impairment [10]. Elevated levels of serum creatinine were seen in cases of renal dysfunction caused by prolonged exposure to glyphosate, suggesting a decline in glomerular filtration. It was reported that serum urea concentrations were unchanged [12]. While other studies documented an elevation in blood creatinine and urea levels, suggesting sub-chronic toxicity, following a 12-week exposure to glyphosate in rats [13]. Additionally, previous studies have indicated that serum creatinine levels serve as a more effective biomarker for nephrotoxicity following exposure to glyphosate [31].

Our findings regarding thyroid functions were in line with those various researchers had previously reported. Perinatal GBH treatment at 5 or 50 mg/kg in Wistar rats produced adult male pups. This study examined how GBPs affect hypothalamic sexual development. De Souza et al. (2017) [32] found that the hypothalamus produced fewer deiodinases, but the pituitary and liver expressed more thyroid hormone receptors. Including a drop in serum TSH, the metabolic profile resembled hypothyroidism in animals. Manservisi et al. (2019) [33] subjected rats to glyphosate, or GBPs, from day 6 of gestation until day 120, drinking water provided 1.75 mg/kg BW/day exposure. Male participants' serum thyroid-stimulating hormone (TSH) levels increased after six weeks of glyphosate exposure (postnatal day 73) and thirteen weeks of glyphosate-based pesticide treatment (postnatal day 125). Hamdaouiet al., (2016) [34] examined the effects of 60 days of oral exposure to 126 mg/kg GBH on bone tissue in Wistar rats, focusing on thyroid hormone modulation. Treatment raised TSH levels, causing osteoporosis in research animals. Adult female mice received 250 mg/kg GBPs by gavage for 7 days, lowering TSH, T3, and T4. Kongtip et al. (2019) [35] found higher TSH, FT3, T3, and T4 levels in conventional farmers than organic farmers.

In this study, the utilization of a natural plant extract, specifically a licorice roots aqueous extract solution, was employed to treat the tissue impairments caused by GBPs. The application of this treatment yielded notable reductions in liver enzymes, urea, creatinine, albumin, and AFP levels within the serum comparing to GBP exposed group. Furthermore, it successfully facilitated the restoration of the histological architecture within the liver that had been previously disrupted by GBP-induced damage. The study conducted by Kou et al., 2020 [36] investigated the impact of glycyrrhizin on liver injury generated by ischemia-reperfusion in rats. The liver ischemia-reperfusion paradigm resulted in a significant increase in serum levels of ALT, AST, lactate dehydrogenase, lipid peroxides in liver tissue, and blood superoxide dismutase activity. The enzymatic activity exhibited an elevation, which was afterwards impeded by the administration of glycyrrhizin. The study demonstrated that isoliquiritigenin effectively alleviated the renal injury induced by cisplatin in mice. Additionally, it was observed that isoliquiritigenin prevented the elevation of serum nitric oxide levels and the occurrence of tissue lipid peroxidation, as reported in a previous study [37]. By a separate investigation, the administration of licorice aqueous extract exhibited a protective effect against hepatotoxicity induced by CCl_4_ in rats. This protective effect was shown through the restoration of normal levels of lipid peroxidation (LPO) and antioxidant enzymes, specifically glutathione (GSH). The potential ability of licorice to mitigate metiram-induced renal damage, as documented by Sakr et al., 2013 [38] could be attributed to its antioxidant characteristics.

The mechanisms behind the preventive effects of glycyrrhizic acid (GA) on non-alcoholic fatty liver disease (NAFLD) induced by a high-fat diet in mice were elucidated by Sun et al., 2017 [39]. Glycyrrhizic acid (GA) is a triterpene glycoside that is found in nature. Following the administration of GA treatment, there was a significant reduction observed in the relative size of the liver, as well as in the activity levels of serum ALT and AST. Additionally, there was a notable decrease in serum lipid levels, as well as blood glucose and insulin levels. The accumulation of lipids in the liver was found to be suppressed by the administration of GA. According to Wang et al. (2016) [40] the administration of GA resulted in a significant decrease in blood levels of liver functional enzymes, namely ALT and AST, as well as inflammatory chemokines. Additionally, GA treatment led to notable improvements in hepatic inflammation and fibrosis, as seen by a reduction in lobular inflammation and pericellular fibrosis. The utilization of *G. glabra* in the treatment of persons suffering from chronic hepatitis was initially observed. The laboratory investigations have demonstrated positive outcomes in terms of liver histology and serum aminotransferases when compared to a placebo [41]. It has been observed that glycyrrhetinic acid exhibits hepatoprotective properties by mitigating oxidative stress and preventing hepatic damage generated by aflatoxin [42]. The administration of a 2 mg/kg body weight dosage of *G. glabra* extract daily has demonstrated a substantial enhancement in liver function among individuals suffering from acute liver diseases [43]. The activity of aspartate aminotransferase is reduced by both aqueous and methanolic extracts of *G. glabra*. This reduction in activity is observed in rats experiencing acute hepatotoxicity induced by CCl_4_ [41].

The progressive decline in AFP levels over a period signifies the process of maturation of immature hepatocytes into their fully developed adult phenotype [44]. AFP serves as an endodermal marker and an early developmental indicator of hepatoblast production. At all-time points, the expression of the early hepatocyte marker AFP was found to be significantly upregulated in mesenchymal stem cells that were treated with GA or 18-glycyrrhetinic acid. The level of expression exhibited a progressive reduction over time [45].

In a study conducted by Mohamed et al., 2019 [46], it was shown that the administration of licorice to rats treated with cadmium chloride resulted in a decrease in the levels of blood AST, ALT, ALP, GGT, and total bilirubin. The available evidence indicates that licorice has the potential to safeguard the structural integrity of the liver against the toxic effects of cadmium (Cd). The active ingredients in licorice, known as glycyrrhizic acid (GL) and glycyrrhetinic acid (GA), have anti-inflammatory and protective properties that can help protect vital organs including the liver, kidneys, heart, and nerves against infections caused by bacteria or viruses [47]. The primary phytoconstituents found in licorice, including flavonoids, glycyrrhetic acid, hydroxycoumarins, and beta-sitosterol, have been observed to possess inhibitory effects on the enzymes involved in the generation of free radicals. The restoration of enzyme levels seen in rats treated with cadmium indicates that licorice may possess a potential protective effect against liver damage generated by cadmium. Polyphenols have an impact on thyroid hormones as well as other hormones, potentially elucidating the reason behind the observed similarity in T4 and T3 levels between rats treated with licorice and Cd and the control group. Several iso-flavonoids can imitate the actions of T3 and T4 hormones, potentially exerting similar effects on the hypothalamus-pituitary feedback loop [48]. It was revealed that licorice consumption resulted in a decrease in T3 levels while having no noticeable impact on T4 levels. However, licorice intake did lead to a considerable increase in TSH levels [49]. The presence of licorice enhanced the magnification of elevated TSH levels induced by the administration of dimethyl nitrosamine (DMN), and additionally caused a significant improvement in the pathological lesions that were created in the thyroid tissue. This improvement was shown by an increase in the size and number of follicles [50]. Besides, the molecular docking assessment is the study of how two or more molecular structures fit into each other. In this study it explored the binding of glyphosate to the binding sites of serum thyroid hormone binding proteins (albumin, thyroxine-binding globulin, and transthyretin). Therefore, glyphosate is bound with thyroid hormone binding proteins instead of thyroid hormones leading to increased levels of free T3 and T4. However, this point needs more investigation to determine if glyphosate affects the synthesis of these carrier proteins.

According to Elmore, (2007) [51] and our study, the light microscopy revealed some hepatocytes in the Glyphosate-treated liver with dark pyknotic nuclei, which are the most recognized signs of apoptosis and these histopathological features have been used for evaluating the liver-protective properties of different elements [52]. The glyphosate-treated liver had positive caspase-3 immunoreactivity. This finding was in concurrence with that of Sevim et al., 2019 [53], and Hashim et al., 2021 [54] who noted a notable increase in the activity of the caspase-3 protein in the liver of treated rats. Our study revealed that TNF-*α* was dramatically increased in the liver of glyphosate-treated rats. These results agree with Zhao et al. (2020) [55] who reported that sustained exposure to lipopolysaccharide leads to activation of Kupffer cells and leads to TNF-*α* production. The minimized caspase-3 expression in hepatic tissues of glyphosate-treated rats followed treated with licorice may be due to the antioxidant activities of licorice. licorice helps to restore the GSH content of the liver and inhibited TNF-*α*secretion [54,56,57]. This study suggests the potential use of licorice, a powerful antioxidant, to reduce the hepatotoxic effects of glyphosate. Our study provides protection against liver damage caused by glyphosate. The results demonstrated the beneficial effects of licorice intervention by showing a reduction in the severity of pathogenic changes. These findings complement those of [54,58]. Besides, the molecular docking approach revealed the affinity of licorice's bioactive compounds to interact with the binding sites of caspase-3, TNFRSF1A, and TNFRSF1B to counteract apoptosis and inflammatory process.

While several interventions have been explored to mitigate glyphosate-based toxicity across various settings. For instance, Chen et al. (2023) demonstrated the efficacy of ultra-high-pressure (UHP) and ferrous sulfate in degrading glyphosate in agricultural soils, indicating potential for soil remediation [15]. In addressing glyphosate contamination in wastewater, a range of methods including physical, biological, and advanced oxidation processes have been investigated. In vivo studies, such as that by Cao et al. (2021), showed that melatonin administration protected against glyphosate-induced oocyte deterioration, suggesting a potential for mitigating adverse reproductive effects [16]. Furthermore, dietary supplementation with black seed was found to improve physiological parameters in animals exposed to glyphosate, highlighting its potential in reducing toxicity [17]. Despite these promising interventions, limitations such as potential environmental impacts and variability in effectiveness across species and different body systems exist. Further studies are needed to elucidate the interplay between these interventions and different systems, such as endocrine disruptors, liver, and kidneys, to fill these gaps in understanding. In our study we examined the GBPs toxic effects and the ameliorative effects of licorice roots aqueous extract solution (100, 200, and 300 mg/mL distilled water, respectively) daily for three weeks. on kidney, liver, thyroid functions, and apoptosis using 50 adult male albino rats. Our results shows that licorice roots aqueous extract solution yielded notable reductions in liver, kidney enzymes, albumin, and AFP levels within the serum. In addition to the immunological tests, including immunohistochemical evaluations of caspase-3 and TNF-*α* expressions, which revealed a dose-dependent attenuation of apoptosis and inflammation with licorice intervention.

We propose that Licorice's hepatoprotective effects have been attributed to its active ingredients, including glycyrrhizic acid (GA) and glycyrrhetinic acid (GA), which possess anti-inflammatory and protective properties [47], flavonoids and glycyrrhetic acid which possess antioxidant activities and isoliquiritigenin which helps in inhibiting tissue lipid peroxidation [37], in addition, licorice bioactive compounds interact with the binding sites of caspase-3, TNFRSF1A, and TNFRSF1B, counteracting apoptosis and inflammatory processes [56], moreover, Licorice's capabilities of inhibiting the TNF-α secretion, contribute to its protective effects against glyphosate-induced tissue damage [56]. Overall, licorice represents a promising natural intervention for ameliorating the toxic effects of glyphosate, offering potential therapeutic strategies for detrimental effect of glyphosate-based pesticide (GBPs), especially because of its affordability due to the extensive cultivation of licorice in the Middle East, Asia, and Europe [58].

Our study's outcome sheds light on potential actions that might be implemented to improve food safety, environmental sustainability, and public health within the broader agricultural settings and regulatory authorities. Since licorice demonstrates efficacy in mitigating the deleterious impacts of GBP residues, it may increase interest in using natural remedies for pesticide detoxification in agricultural settings. This might impact agricultural practices by encouraging farmers to use other approaches to reducing pesticide residues, such adding licorice extracts to soil and livestock feed. Furthermore, this will encourage further research into similar natural compounds that exhibit similar detoxifying properties, thus promoting the use of sustainable and eco-friendly agriculture methods. Moreover, these findings could affect risk assessment and pesticide control strategies, as regulatory authorities may integrate licorice or similar compounds that demonstrate efficacy in reducing the adverse effects of pesticides into pesticide management programs or enforce restrictions on the levels of GBP residues in agricultural goods.

There are a few noteworthy limitations to the study. The inability to determine the precise active principles in licorice that mitigate the detrimental effects of glyphosate limits the study's ability to adequately explain the protective properties of licorice. Ultimately, although molecular docking provides valuable insights, further experimental validation is necessary to fully understand the mechanisms behind the ameliorate impacts of licorice roots aqueous extract on glyphosate-induced tissue damage.

## 5 Conclusions

Using rats as an experimental animal model, the current study found that administration of GBPs caused hepatotoxicity, renal toxicity, and hypothyroidism, while extracts of licorice had therapeutic benefits on the measured parameters. Furthermore, GBPs generated degenerative histological structures in liver tissue, which were reversed when licorice extract was administered. It appears that greater doses of licorice root extract (300 mg/mL DW) may have synergistic activity and offer the highest protective effects. The serum enzymes generated by licorice solution heal at a rate proportional to the therapy length, after three weeks of continuous administration, the herbs have almost completely restored normal function. In conclusion, our study sheds the light on the potential of licorice bioactive ingredients as a cornerstone for therapeutic applications. By focusing on its key components, we aim to further investigate its efficacy and safety profile through future investigations into its toxicity. Specifically, we intend to compare the formation and active principle separately, shedding light on its mechanisms of action and potential benefits. Furthermore, our research addresses the urgent need to assess the residual effects of glyphosate pesticide on both aromatic and medicinal plants, with a hypothesis suggesting that licorice root extract may play a pivotal role in mitigating pesticide residues. This not only holds promise for improving the quality and value of agricultural produce for international exportation but also emphasizes the potential for licorice extract to be used as a natural remedy for pesticide detoxification in a variety of agricultural settings. Through future studies, we aim to fully uncover the potential of licorice extract, both in agricultural applications and beyond, paving the way for safer and more sustainable plant-based therapeutic practices.

## Consent to participate

All authors whose names appear on the submission made substantial contributions to the conception or design of the work; or the acquisition, analysis, or interpretation of data used in this work.

## Funding

This study was supported by Princess 10.13039/501100004242Nourah bint Abdulrahman University Researchers Supporting Project number (PNURSP2024R30), Princess 10.13039/501100004242Nourah bint Abdulrahman University, Riyadh, Saudi Arabia. This research was funded by the Researchers Supporting Project number (RSPD2024R811), King Saud University, Riyadh, Saudi Arabia.

## Data availability

The data associated with this study will be available on request.

## CRediT authorship contribution statement

**Ahmed N. Elkattan:** Methodology, Data curation, Conceptualization. **Sayed El-saadany:** Supervision, Conceptualization. **Mohamed Azzazy:** Supervision, Conceptualization. **Tarek M. Okda:** Supervision, Conceptualization. **Maha Mamdouh:** Software. **Osama Ahmed:** Methodology. **Ali H. El-Far:** Software, Methodology. **Manar ElKhayat:** Writing – review & editing, Visualization, Validation. **Ghadeer M. Albadrani:** Funding acquisition. **Muath Q. Al-Ghadi:** Funding acquisition. **Mohamed M. Abdel-Daim:** Funding acquisition. **Hala El Daous:** Writing – review & editing, Writing – original draft, Methodology.

## Declaration of competing interest

The authors declare that they have no known competing financial interests or personal relationships that could have appeared to influence the work reported in this paper.
